# Effect of temperature and preservative selection on C-peptide degradation in randomly voided urine

**DOI:** 10.1016/j.jcte.2026.100438

**Published:** 2026-04-01

**Authors:** Eli H. Hagedorn, Cameron R. Rostron, Anthony Acton, Mallory Oswalt, Robert V. Considine, Carmella Evans-Molina

**Affiliations:** a Departments of Biochemistry and Molecular Biology, Indiana University School of Medicine, Indianapolis, IN 46202, USA; b Departments of Anatomy, Cell Biology, and Physiology, Indiana University School of Medicine, Indianapolis, IN 46202, USA; c Departments of Medicine, Indiana University School of Medicine, Indianapolis, IN 46202, USA; d Pediatrics Indiana University School of Medicine, Indianapolis, IN 46202, USA; e Center for Diabetes and Metabolic Diseases, Indiana University School of Medicine, Indianapolis, IN 46202, USA; f Herman B Wells Center for Pediatric Research, Indiana University School of Medicine, Indianapolis, IN 46202, USA; g Roudebush VA Medical Center, Indianapolis, IN 46202, USA

**Keywords:** Urine C-Peptide, Pre-analytical stability, Specimen handling, Type 1 diabetes, β cell function, Biomarker preservation

## Abstract

**Background:**

Type 1 diabetes (T1D) results in the loss of endogenous production of insulin and C-peptide, which are co-secreted by pancreatic β cells. Serum C-peptide following mixed-meal tolerance tests is commonly used in T1D trials to assess residual insulin secretion. Urine C-peptide (UCP) offers a non-invasive alternative suitable for home collection. Here, we aimed to optimize urine handling practices to support remote urine C-peptide monitoring in clinical trial settings.

**Methods:**

Randomly-voided urine was collected from 18 non-diabetic participants (mean age: 46.6 years; 94.4% female). Samples were treated with no preservative (NP), boric acid (BA), or sodium carbonate (SC) and incubated at 4°C or room temperature (RT, 22°C) for up to 72 h. Use of cold packs after initial refrigeration was also tested. UCP was quantified using the TOSOH immunoenzymatic assay and normalized to urine creatinine (UCP:Cr). Stability was expressed as percent change from baseline (%UCP:Cr).

**Results:**

NP samples lost %UCP:Cr more rapidly at RT, with decreases at 48 h (−16%, P < 0.05) and 72 h (−21%, P = 0.02) compared to 4°C. BA-preserved samples had declined %UCP:Cr at 72 h (−16%; P = 0.04) compared to 4°C. SC maintained UCP:Cr stability, showing no significant change in %UCP:Cr between 4°C and RT. Samples kept on cold packs following initial refrigeration showed no significant loss in %UCP:Cr compared to samples continuously refrigerated for 72 h.

**Conclusions:**

Cold storage of urine slows UCP:Cr degradation, and SC stabilizes UCP:Cr at room temperature. Our findings suggest that UCP:Cr offers a practical option for at-home urine collection in clinical studies.

## Highlights

• Urine C-peptide (UCP) is a biomarker of β cell function that is susceptible to degradation.
• Sodium carbonate (SC) preserves UCP:creatinine ratio (UCP:Cr) for up to 72 hours.
• Hybrid storage using refrigeration followed by cold-pack transport maintains UCP stability.
• Omission of centrifugation markedly accelerates UCP degradation.
• These findings provide guidance for remote urine collection and shipment to improve biomarker reliability in clinical trials.

## Introduction

Insulin, a critical peptide hormone produced and secreted by pancreatic β cells, regulates blood glucose levels by promoting glucose uptake into peripheral tissues [1]. In type 1 diabetes (T1D), progressive autoimmune destruction of β cells leads to decreased insulin production and near complete insulin deficiency [2]. Accurate assessment of endogenous insulin production is vital for several aspects of diabetes management and diagnosis, including monitoring of T1D risk and disease progression, determining the efficacy of therapeutic interventions seeking to slow the loss of functional β cells in those with and at-risk of T1D [3], and distinguishing T1D from type 2 diabetes, where β cell function is typically higher. However, measurement of insulin secretion is complicated by exogenous insulin therapy and anti-insulin antibodies [4] that can interfere with insulin immunoassays. C-peptide, which is cleaved from proinsulin, is secreted in equimolar amounts to insulin [5] and reliably quantifies endogenous insulin secretion [6] while bypassing the limitations of direct insulin quantification in T1D. C-peptide has become a widely-used and valuable biomarker in T1D. In clinical trials, area under the curve of C-peptide (C-peptide AUC) secreted in response to a mixed-meal tolerance test (MMTT) serves as the gold standard for assessing β cell function [7] and the efficacy of experimental therapies [8], [9], [10].

The process of preparing for and undergoing a MMTT is time-consuming and laborious for both trial participants and clinical research staff [11]. Validated single timepoint alternatives to a MMTT include unstimulated or glucagon-stimulated measurement of serum C-peptide [5]; however, these methods also require a clinical setting, and C-peptide collected in blood shows degradation by 24 h when collected in standard conditions [12]. In lieu of serum assays, urine C-peptide (UCP) measurement has emerged as a non-invasive alternative that does not require venipuncture or collection in a hospital setting [13]. Several studies have illustrated that measurement of single-void UCP to creatinine ratio (UCP:Cr) is a stable biomarker for the long-term assessment of β cell function [14], [15], [16], [17], [18], [19], [20], [21] and may serve as a potential replacement for the MMTT [16]. Only one previous paper [15], which utilized a C-peptide assay that is not typically used in T1D clinical trials and exhibits substantial proinsulin cross-reactivity, has tested different handling practices for UCP. Here, we determined optimal sample collection and storage conditions for remote monitoring of UCP:Cr levels using the TOSOH AIA II C-peptide assay, which is the preferred assay for several clinical trial consortia including the Type 1 Diabetes TrialNet and the Immune Tolerance Network and has minimal proinsulin cross-reactivity.

## Methods

### Demographics and sample acquisition

The study protocol was approved by the Indiana University Institutional Review Board (IRB protocol #1411938757). Randomly voided urine samples were collected from two non-diabetic cohorts to ensure measurable levels of C-peptide. Cohort 1 included 18 non-diabetic adults (mean age, 46.6 years; range 22.2–63.8 years; 94.4% female) and cohort 2 included 10 non-diabetic adults (mean age, 51.2 years; range 36–62 years; 100% female). Following collection, samples were transported from the clinical facility to the lab in an isothermal container without preservatives for immediate processing.

### Sample treatment and storage

Urine samples from cohort 1 were centrifuged for 5 min at 259 rcf to remove debris prior to chemical treatment. Samples from cohort 2 were not centrifuged and were immediately treated with preservatives in standard urine collection cups. The mean time from sample collection to aliquoting was 3.48 h (range 2.57–4.80 h). Following centrifugation, urine samples were aliquoted into sterile, labeled tubes containing boric acid (BA, Sigma-Alrich #B0252; 215 mM), sodium carbonate (SC, Thermo Scientific #206800010; 80 mM), or no preservative (NP). Immediately following chemical treatment, the baseline (0 h) aliquots were collected and frozen at −80°C until analysis. The remaining aliquots were stored at room temperature (RT, 22°C) or refrigerated (4°C) for up to 72 h. After 24 h, a subset of refrigerated samples was transferred to insulated polystyrene foam shipping containers with cold packs. To simulate shipping conditions, the cold packs were not replaced during the study. These containers were kept at ambient temperature (22°C) on a reciprocal shaker to simulate movement that would occur during shipping. Aliquots from each sample condition were collected at 0, 12, 24, 48, and 72 h and immediately frozen at −80°C until analysis. Previous studies have shown that C-peptide concentration remains stable for at least 4 months of storage in samples maintained at this temperature [15].

*C-Peptide Assay*.

UCP concentrations were measured using a two-site immunoenzymatic assay (ST AIA-PACK C-Peptide II; TOSOH Bioscience, South San Francisco, CA) on the TOSOH AIA-360 automated immunoassay analyzer. The assay has a sensitivity of 0.008 ng/mL, a proinsulin cross-reactivity of 0.047%, and an undetectable cross-reactivity with insulin, per the manufacturer. The manufacturer has also tested the precision of this assay in urine samples, reporting a coefficient of variation of 4.4% (n = 20).

To account for interindividual differences in urine concentration, C-peptide levels were normalized to creatinine and expressed as UCP:Cr. Creatinine normalization is the standard approach for spot urine collections as urine creatinine levels are stable in a majority of incubation temperatures [22]. Creatinine concentration (mg/dL) was measured using the Jaffe method on the Roche Integra 400 Plus analyzer (Roche Diagnostics, Indianapolis, IN) and was found to remain stable in urine samples throughout the study. The percentage change in UCP:Cr at each time point relative to baseline (%UCP:Cr) was calculated to estimate C-peptide degradation. All assays were performed at the Translation Core of the Indiana Diabetes Research Center at Indiana University School of Medicine (Indianapolis, IN).

### Data analysis

Data were analyzed using two-way repeated-measures analysis of variance (RM ANOVA) to assess the effects of preservative type or incubation temperature on UCP:Cr over time. When RM ANOVA analysis was not possible, mixed-effects analysis was performed to assess the effects of preservative type and incubation conditions on UCP:Cr over time. Post hoc comparisons were conducted using Tukey’s multiple comparisons test. Statistical significance was defined as *p* < 0.05. All statistical analyses were performed using GraphPad Prism (Version 10.4.2).

## Results

### UCP:Cr stability is influenced by preservatives and temperature

**Fig. 1:**
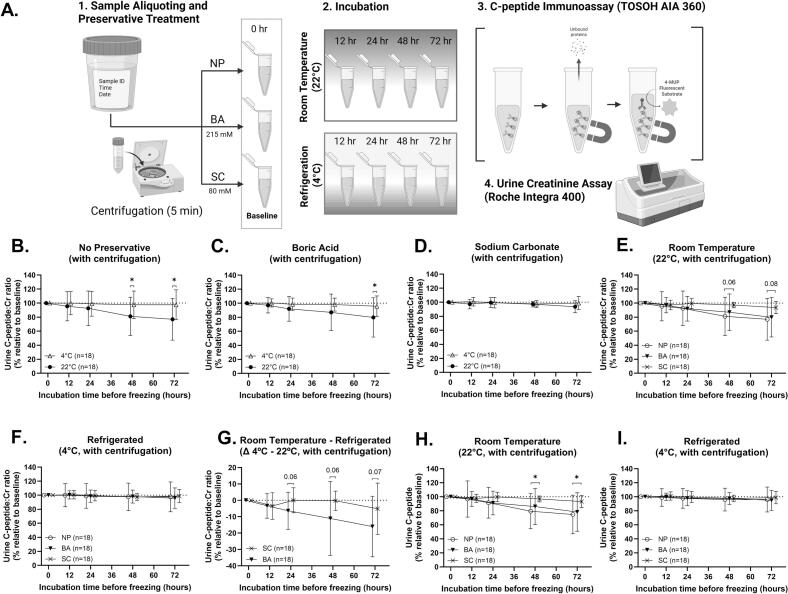
Stability of UCP:Cr Varies by Preservative and Temperature. (A) Experimental design: centrifuged urine samples were treated with no preservative (NP), boric acid (BA), or sodium carbonate (SC) and incubated at room temperature (22°C) or under refrigeration (4°C) for 72 h. %UCP:Cr is compared over time by preservative (B-D) and temperature (E-F). (G) Difference in %UCP:Cr between temperatures in preserved samples. (H-I) %UCP (without normalization to creatinine) over time by temperature. Presented as mean ± SD; analyzed using two-way RM-ANOVA with Tukey’s post-hoc. *p < 0.05.

To assess the short-term stability of UCP:Cr, urine from 18 non-diabetic individuals was stored with NP, BA, or SC and incubated for 12, 24, 48, or 72 h at either room temperature (RT, 22°C) or with refrigeration (4°C) (Fig. 1**A**). Percent change in UCP:Cr (%UCP:Cr) was calculated relative to the 0-hour baseline UCP:Cr. Both preservative and temperature significantly affected %UCP:Cr over time. At early time points (12–24 h), %UCP:Cr did not differ between RT and refrigerated conditions, indicating initial stability independent of preservatives (Fig. 1**B–D**). However, with longer incubation, NP-treated RT samples showed a mean %UCP:Cr change of −16.43% (–32.57% to −0.2848% 95% CI, P < 0.05) at 48 h and −20.70% (−35.60% to −5.803 95% CI, P = 0.02) at 72 h (Fig. 1**B**). BA-treated RT samples showed a mean %UCP:Cr change of −11.07% (−24.59% to 2.455% 95% CI, P = 0.10) at 48 h and −16.01% at 72 h (−31.32% to −0.7081% 95% CI, *P* = 0.0410) (Fig. 1**C**). In contrast, SC-treated RT samples exhibited no significant changes in %UCP:Cr throughout the 72-hour incubation (Fig. 1**D**). Additionally, the coefficient of variation (%CV) for %UCP:Cr was lowest in SC-preserved urine (Table 1), indicating greater stability and consistency across samples with SC treatment.

**Table 1:** Stability of urine C-peptide to creatinine ratio (%UCP:Cr) over time under different preservation and temperature conditions.

		No preservative (n = 18)			Boric acid (n = 18)			Sodium carbonate (n = 18)		
	Hours	Mean	SD	%CV	Mean	SD	%CV	Mean	SD	%CV
RT	12	95.51	20.81	21.79%	97.02	10.81	11.14%	97.33	6.741	6.926%
	24	92.6	24.61	26.58%	92	17.59	19.12%	99.4	7.194	7.237%
	48	81.16	27.25	33.57%	87	26.01	29.89%	97.24	3.988	4.101%
	72	76.95	29.71	38.61%	79.69	28.01	35.14%	93.67	8.653	9.238%
4°C	12	99.96	16.48	16.49%	100.4	5.896	5.874%	100.8	6.082	6.036%
	24	98.97	17.61	17.79%	98.39	9.004	9.151%	99.38	7.537	7.584%
	48	97.59	19.62	20.11%	98.07	9.246	9.429%	97.51	4.966	5.093%
	72	97.65	21.34	21.85%	95.7	14.57	15.22%	98.81	9.431	9.544%

Values represent mean ± standard deviation (SD) of percent change in urine C-peptide to creatinine ratio (%UCP:Cr) in centrifuged urine samples relative to baseline over 72 h. %CV = coefficient of variation. RT = room temperature (22°C). UCP:Cr = urine C-peptide to creatinine ratio.

### SC treatment maintains UCP:Cr stability compared to NP and BA

We next compared preservative treatments in the RT samples. After 72 h, SC treatment showed a trend toward improved stability compared with BA (–13.98%, −31.45% to 3.483% 95% CI, *P* = 0.13) and NP (–16.10%, −35.18 to 1.745 95% CI, *P* = 0.08) (Fig. 1**E**). Under refrigeration, no significant differences were detected within or between treatment groups across time points (Fig. 1**F**), indicating that refrigeration alone adequately preserves UCP:Cr. To evaluate the differential effect of temperature on UCP:Cr preservation in each sample, %UCP:Cr at RT was subtracted from paired refrigerated measurements (Fig. 1**G)**. This analysis showed a consistent trend toward improved stability with SC treatment compared to BA at 24 h (−6.42%, −13.00% to 0.1714% 95% CI, *P* = 0.0559), 48 h (−10.80%, −22.30% to 0.6907% 95% CI, *P* = 0.0640), and 72 h (−10.87%, −22.49% to 0.7512% 95% CI, *P* = 0.0658); however, these changes did not reach statistical significance. Additionally, we examined changes in UCP concentrations (%UCP) without normalization to creatinine at RT (Fig. 1**H**) and with refrigeration (Fig. 1**I**). At both temperatures, %UCP showed similar patterns of degradation compared to evaluation of %UCP:Cr, strengthening the internal validity of the UCP:Cr measurement. At RT, SC treatment improved %UCP compared to NP at 48 h (−17.49%, −32.80% to −2.183% 95% CI, *P* = 0.0238) and 72 h (−18.43%, −35.69% to −1.184% 95% CI, *P* = 0.0349) (Fig. 1**H**).

### Storage in isothermal containers preserves UCP:Cr similarly to refrigeration

**Fig. 2:**
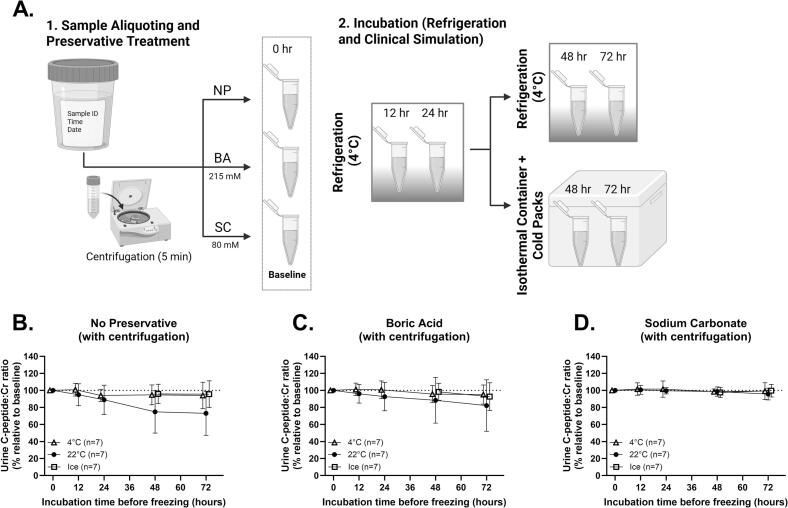
Isothermal Storage on Ice Preserves UCP:Cr Stability After Initial Refrigeration. (A) Experimental design: centrifuged urine samples were treated with no preservative (NP), boric acid (BA), or sodium carbonate (SC), incubated under refrigeration (4°C) for 24 h, and then refrigerated or transferred to polystyrene containers with cold packs (Ice) for 48 h. (B-D) %UCP:Cr over time comparing refrigeration (4°C), room temperature (22°C), and cold packs (Ice). Presented as mean ± SD; analyzed by mixed-effects model.

To evaluate alternative storage conditions for at-home collection of urine, we tested a hybrid protocol where urine was refrigerated for 24 h, then moved to insulated polystyrene foam containers with a cold pack (isothermal storage condition) for the remaining 48 h (Fig. 2**A**). In urine from 7 non-diabetic volunteers from cohort 1 (mean age, 50.7 years; range, 22.2–63.8 years; 100% female), UCP:Cr levels in isothermal storage showed no significant difference compared with refrigeration across NP, BA, and SC groups (Fig. 2**B–D**). The similarity of %UCP:Cr values between samples stored in isothermal containers and refrigeration suggest that these methods are interchangeable. These results support the use of isothermal containers with cold packs in large-scale trials to reduce UCP:Cr loss that may result from in-home collection and shipment conditions.

### Absence of urine centrifugation impairs UCP:Cr stability

**Fig. 3:**
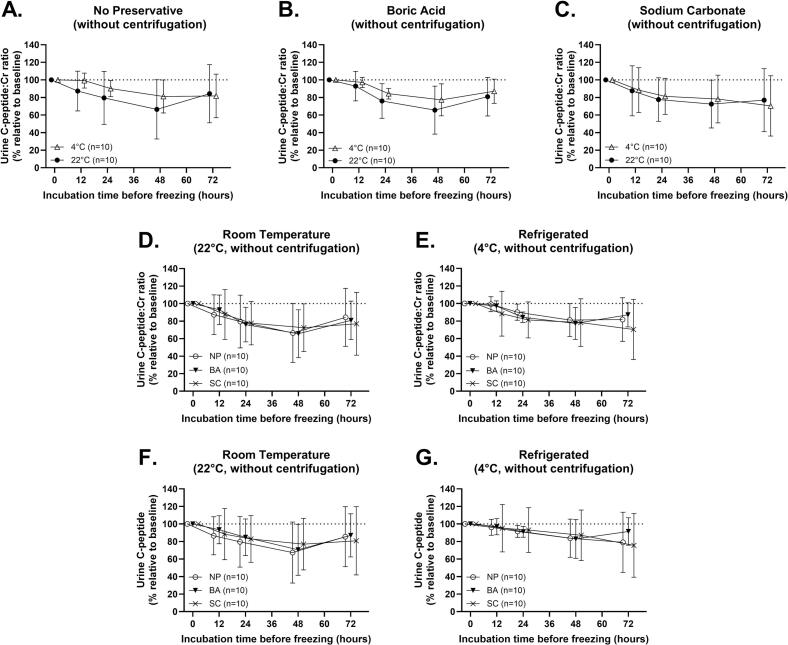
Lack of Centrifugation Before Treatment Exacerbates UCP:Cr Degradation. Percent change in UCP:Cr from baseline in non-centrifuged urine samples. Samples were treated with no preservative (NP), boric acid (BA), or sodium carbonate (SC), then incubated at room temperature (22°C) or under refrigeration (4°C) for 72 h. %UCP:Cr is compared by preservative (A-C) and temperature (D-E) over time. (F-G) %UCP (without normalization to creatinine) over time by temperature. Presented as mean ± SD; analyzed using two-way RM-ANOVA with Tukey’s post-hoc.

Finally, we investigated how omitting centrifugation, which could be delayed during at-home collection, affects UCP:Cr stability. Urine samples from a second cohort of 10 non-diabetic individuals (mean age, 51.2 years; range, 36–62 years; 100% female) were treated identically with NP, BA, or SC, but did not undergo centrifugation prior to incubation. Treated samples were incubated at 22°C or 4°C with aliquots taken at 0, 12, 24, 48, and 72 h. Across treatments, there was substantial loss of UCP:Cr in non-centrifuged samples. The mean %UCP:Cr of all RT samples decreased by 22.30% (−26.65% to −17.95%, 95% CI) at 24 h, 31.78% (−41.07% to −22.50%, 95% CI) at 48 h, and 19.27% (−28.39% to −10.16%, 95% CI) at 72 h. Similarly, the mean %UCP:Cr of all refrigerated samples decreased by 14.76% (−25.89% to −3.64%, 95% CI) at 24 h, 21.14% (−26.09% to −16.20%, 95% CI) at 48 h, and 20.28% (−41.47% to 0.90%, 95% CI) at 72 h. No significant differences in %UCP:Cr were detected between RT samples and refrigerated samples (Fig. 3**A–C**) or among preservatives (Fig. 3**D and E**). We assessed changes in %UCP without creatinine normalization to control for any changes in creatinine. At both RT and with refrigeration (Fig. 3F-G), %UCP mirrored the degradation patterns of %UCP:Cr (Fig. 3**F and G**). These results suggest that centrifugation is a critical pre-analytical step for maintaining UCP:Cr stability and its omission may limit the stability of UCP:Cr levels, even with favorable incubation and preservation conditions.

## Discussion

In this study, we systematically evaluated pre-analytical factors that influence UCP:Cr stability, focusing on preservative selection, storage temperature, and centrifugation, to inform best practices for remote analyte collection. Several studies have established UCP:Cr as a noninvasive biomarker of endogenous insulin secretion in T1D [14], [16], [20], type 2 diabetes [14], [18], [21], pancreatic islet allografts [23], and maturity-onset diabetes of the young [24]. Recent T1D immunomodulatory clinical trials have relied on stimulated serum C-peptide assays to assess β cell function in trial participants [10], [25], [26]; however, these tests are labor-intensive, require frequent clinic visits, and can be challenging in very young participants [27], thus limiting participant retention and geographic reach. Although UCP:Cr is less sensitive than serum C-peptide assays for detecting early loss of β cell function [20], it remains valuable for long-term follow-up studies, especially when evaluating whether residual C-peptide is present or absent. Moreover, the simplicity of urine collection and the stability of urine analytes make UCP:Cr particularly attractive for clinical trials requiring remote or frequent monitoring. Validation of methods to optimize home collection of UCP:Cr is especially relevant due to the development of immune modifying therapies like teplizumab, which delay the onset of T1D and preserve β cell function [25], [26]. However, concerns about variable sample collection and analyte processing have hindered the widespread use of UCP:Cr measurements in clinical studies [5]. Our findings identify several practical strategies to mitigate pre-analytical degradation and strengthen the reliability of UCP:Cr in future studies.

We first compared chemical urine preservatives that could easily be applied to practical in-home collections for clinical studies. BA is one of the most common preservatives for clinical urine collection, and SC can stabilize peptides prone to rapid degradation [28], [29]. We observed that SC preservation consistently maintained UCP:Cr stability over 72 h, while BA offered partial protection up to 48 h. UCP:Cr in samples without preservatives was only stable for up to 24 h at room temperature. This finding suggests that SC might be a superior preservative for UCP:Cr compared to BA, which is notable given that BA is the preservative of choice for urine collections [30]. BA effectively delays bacterial contamination at room temperature and 4°C [31], and the only other study evaluating UCP:Cr stability reported equivalent performance of BA and SC for up to 72 h [15]. However, not all studies have found BA to be sufficient for preserving peptide or protein stability. Proteomic analyses showed that BA treatment was insufficient to prevent urine protein degradation at room temperature [32], and a study of UCP in 24 h urine collection showed that SC improved stability at room temperature [29]; however, this study did not compare the effects of BA preservation. Overall, the data presented here suggest that the use of SC as a preservative may be preferable in UCP assays.

Differences in reported preservative performance may reflect variation in the C-peptide immunoassays employed. Since C-peptide is a component of uncleaved proinsulin, C-peptide assays can cross-react with proinsulin, and current standards recommend using assays with <10% proinsulin cross-reactivity [5]. In their study, McDonald *et al.* [15] utilized the Roche E170, a common C-peptide immunoassay, which exhibits high proinsulin cross-reactivity (31.5%, per manufacturer). In this manuscript, we utilized the TOSOH C-peptide automated immunoassay, which is preferred by several networks, including the Type 1 Diabetes TrialNet, and has a markedly lower proinsulin cross-reactivity (0.047%, per manufacturer). This distinction is clinically relevant because elevated serum proinsulin-to-C-peptide ratios predict progression to T1D among autoantibody-positive individuals [33], and proinsulin levels persist in >95% of individuals with long-standing T1D despite undetectable C-peptide [34]. Consequently, C-peptide assays with high proinsulin cross-reactivity could overestimate β cell function. These findings emphasize that accurate quantification of β cell function using UCP:Cr requires both highly specific assays and optimized sample handling.

In addition to the preservative effects, we confirmed that refrigeration (4°C) enhanced UCP:Cr stability across all treatments, consistent with prior reports [15]. We expanded on these findings by testing a hybrid protocol in which samples were refrigerated for 24 h prior to storage on cold packs in insulated polystyrene containers. This approach maintained UCP:Cr integrity for 72 h, replicating the benefits of refrigeration and simulating realistic home-collection conditions. Notably, these findings are limited to containers kept in a climate-controlled laboratory. It is likely that more extreme external conditions such as intense heat or cold might more drastically impact UCP:Cr stability and should be considered in future studies.

Importantly, in our study, the omission of centrifugation in urine samples exacerbated apparent UCP:Cr degradation across all treatments. UCP:Cr measurement feasibility has been tested in laboratory and hospital settings [35], but the impact of uncentrifuged urine on C-peptide degradation has not been examined. One explanation for this degradation could be remnant urinary proteases in uncentrifuged urine, a typical component of antimicrobial protection that increases risk of peptide digestion. Ogawa *et al.* demonstrated that SC stabilizes UCP primarily by urine alkalinization, which inhibits acid-activated proteases such as trypsin [29]. Similar degradation mechanisms have been reported for other urinary proteins including albumin, which undergoes fragmentation during storage [36], [37] and pepsin-mediated digestion in acidic conditions [38]. However, our uncentrifuged dataset showed UCP:Cr degradation even with alkaline preservatives, suggesting that additional measures might be needed to ensure stability. Bacterial contamination also accelerates peptide degradation, especially in uncentrifuged urine samples where cellular debris provides a nutrient sources for microbes [39]. Previous studies have shown that urine centrifugation can prevent bacterial overgrowth and subsequent proteome changes, especially when stored at room temperature [39]. BA is typically an effective bacteriostatic, but our data indicates it may be insufficient to prevent UCP:Cr loss. If UCP:Cr is to be measured in home-collected urine samples, easily accessible alternatives to centrifugation may need to be implemented.

These limitations highlight the need for practical alternatives to centrifugation that can be implemented in decentralized or home-based collection workflows. The DEFEND-2 T1D intervention trial measured UCP:Cr in urine samples that were frozen without preservatives or centrifugation prior to shipment to a central laboratory [40]. While immediate freezing may be a practical substitute for pre-centrifugation, evidence indicates it may not fully preserve sample integrity. Bernini *et al.* (2011) reported that even with freezing, urine with particulate matter had alterations in the metabolic profile compared to particulate-free samples [41]. Importantly, this study concluded that either mild centrifugation or filtration with a 0.20 µm filter prior to freezing urine samples was sufficient to reduce sample degradation and decrease changes to the urine metabolic profile [41]. Importantly, in this study they concluded that either mild centrifugation or filtration with a 0.20 µm filter prior to freezing urine samples was sufficient to reduce sample degradation and decrease changes to the urine metabolic profile [41]. Given the logistical barriers associated with centrifugation in decentralized settings, single-use filtration devices may represent a more scalable and accessible strategy for preserving UCP:Cr stability and warrant further investigation.

This study had a small sample size and was restricted to non-diabetic individuals. However, a strength of our study is that the centrifuged samples utilized here had a wide range of initial UCP:Cr levels from 0.16 to 12.83 nmol/mmol. This strengthens the generalizability of our findings to individuals with early onset T1D, as the DEFEND-2 study reported average UCP:Cr ranges from 6-8 nmol/mmol in the first year of disease [40], and UCP:Cr values are usually under 0.2 nmol/mmol in long-standing T1D [24]. It is possible that higher levels of UCP:Cr variation might be observed in samples with lower baseline levels of UCP, which would be expected in individuals with diminished β cell function. The vast majority of subjects in this study were female, which could lead to a higher average UCP:Cr, as previous work has shown that female subjects have a 1.48 fold higher UCP:Cr due to lower urine creatinine levels [42]. Future work should validate these findings in diabetic cohorts with a more balanced sex distribution and explore long-term stability beyond 72 h. Future clinical studies should also consider the effects of urine filtration as it compares to centrifugation, as filtration could be a pragmatic solution to prevent UCP:Cr degradation in the absence of access to a centrifuge in the home-collection scenario. Notwithstanding these limitations, our results have practical implications for multi-site studies and clinical trials implementing noninvasive β cell function testing where at-home collection is desirable.

## Conclusion

In summary, our findings demonstrate that pre-analytical factors are key determinants of UCP:Cr stability and can be substantially optimized through practical, low-cost interventions. The combined use of appropriate chemical preservation, cold storage, and strategies to reduce particulate-associated degradation offers a feasible framework for improving sample integrity in decentralized and home-based collection settings. These methodological considerations are particularly relevant as UCP:Cr is increasingly applied in clinical trials and longitudinal studies of β cell function. While further validation in individuals with diabetes and under real-world environmental conditions is warranted, implementation of standardized pre-analytical protocols has the potential to enhance the reliability, scalability, and clinical utility of UCP:Cr as a noninvasive biomarker.

## CRediT authorship contribution statement

**Eli H. Hagedorn:** Writing – review & editing, Writing – original draft, Methodology, Investigation, Formal analysis, Conceptualization. **Cameron R. Rostron:** Writing – review & editing, Methodology, Investigation, Formal analysis, Conceptualization. **Anthony Acton:** Writing – review & editing, Investigation. **Mallory Oswalt:** Writing – review & editing, Investigation. **Robert V. Considine:** Writing – review & editing, Supervision, Resources, Methodology. **Carmella Evans-Molina:** Writing – review & editing, Writing – original draft, Supervision, Methodology, Investigation, Funding acquisition, Conceptualization.

## Declaration of competing interest

The authors declare the following financial interests/personal relationships which may be considered as potential competing interests: We acknowledge the following competing interests: CEM has served on advisory boards related to T1D research clinical trial initiatives: Isla Technologies, DiogenyX, Neurodon, Sanofi; In-kind research support from BMS (access to compounds for a JDRF funded project); past Investigator initiated grants: Lilly Pharmaceuticals and Astellas Pharmaceuticals. CEM has a patent (16/291,668) for Extracellular Vesicle Ribonucleic Acid (RNA) Cargo as a Biomarker of Hyperglycemia and Type 1 Diabetes, and CEM has a provisional patent (63/285,765) Biomarker for Type 1 Diabetes (PDIA1 as a biomarker of β cell stress). These activities have not dealt directly with topics covered in this manuscript.

## Data Availability

Data presented in this manuscript are available from the corresponding author upon request.
